# Immune Thrombotic Thrombocytopenic Purpura Presenting With Transient Neurological Deficits: A Case Report

**DOI:** 10.1155/crh/4650998

**Published:** 2026-11-09

**Authors:** Hisham Eltayeb Ahmed Elhasan, Tarig Merghani, Fathelrahman Ahmed, Syed Majid Hamid, Azza Alawad

**Affiliations:** ^1^ Department of Medicine Gargash Hospital Dubai UAE duhs.edu.pk; ^2^ Department of Physiology RAK Medical and Health Sciences University Ras Al-Khaimah UAE; ^3^ Department of Endocrinology and Diabetes Ibrahim Bin Hamad Obeidallah Hospital Ras Al-Khaimah UAE; ^4^ Department of Physiology Al-Neelain University Khartoum Sudan neelain.edu.sd

**Keywords:** ADAMTS13 protein, hemolytic anemia, microangiopathic, thrombocytopenia, thrombocytopenic purpura

## Abstract

**Background:**

Immune thrombotic thrombocytopenic purpura (iTTP) is a rare but fatal disorder characterized by microangiopathic hemolytic anemia, severe thrombocytopenia, and ischemic damage of organs due to widespread platelet‐rich thrombi in small blood vessels. Early detection and prompt treatment are crucial to reducing mortality.

**Case Presentation:**

We report a case of a female, 66‐year‐old, who presented with transient episode of left‐sided weakness and aphasia that resolved within 30 min. Initial neuroimaging showed no evidence of ischemia. Laboratory evaluation showed severe thrombocytopenia, severe anemia, high lactate dehydrogenase (LDH), reticulocytosis, and the presence of schistocytes in peripheral blood. The calculated PLASMIC score was 6, and ADAMTS13 activity showed severe deficiency, supporting the diagnosis of iTTP. The patient responded well to corticosteroids and therapeutic plasma exchange, with significant clinical and hematological improvement following the treatment. She was discharged after 2 weeks in good condition with a plan for regular follow‐up.

**Conclusion:**

This case emphasizes the significance of including iTTP in the differential diagnosis of transient neurological attacks in patients with hematological abnormalities. Early initiation of plasma exchange and immunosuppressive therapy are essential to prevent organ damage and further complications.

## 1. Introduction

Immune thrombotic thrombocytopenic purpura (iTTP) is an uncommon hematologic emergency that affects about 2–6 patients out of one million subjects annually [1]. It is characterized by thrombosis, low platelet counts, and microangiopathic hemolytic anemia (MAHA). Systemic manifestations including renal and neurological findings are recognized associations. The condition is primarily caused by reduction of ADAMTS13 (a disintegrin and metalloprotease with thrombospondin Type 1 motif, Member 13) activity, which is responsible for cleaving ultralarge von Willebrand factor (vWF) multimers [2]. Severe ADAMTS13 deficiency (< 10% activity) results in widespread platelet aggregation and microvascular thrombosis through accumulation of large vWF multimers [3]. Reduction of ADAMTS13 may result from genetic mutation causing reduced production (congenital TTP) or more commonly due to autoantibodies (iTTP).

Clinical presentation of iTTP includes a wide range of symptoms such as fatigue, dyspnea, petechiae, neurological deficits, and organ dysfunction. The diagnosis often relies on laboratory findings as the classical five features (fever, MAHA, thrombocytopenia, renal dysfunction, and neurological symptoms) may not be all evisent at presentation [4]. The PLASMIC score is utilized clinically in estimating the likelihood of severe ADAMTS13 deficiency and may guide early treatment decisions [5, 6].

## 2. Case Presentation

A 66‐year‐old female was brought to the emergency department at Gargash Hospital, Dubai, UAE, in July 2025 with sudden aphasia and left‐sided weakness. Before reaching the hospital, the neurological deficits were resolved spontaneously, with a total duration of approximately 30 min. She was initially admitted as a case of transient ischemic attack (TIA). On examination, she was conscious and oriented (Glasgow Coma Scale score of 15/15). Neurological examination was unremarkable, with normal tone, power, reflexes, and intact sensation. However, general examination revealed pallor and multiple ecchymotic patches over the trunk and limbs. Cardiovascular, respiratory, and abdominal examinations were unremarkable.

Initial laboratory investigations (Table 1) showed severe anemia (hemoglobin 7.8 g/dL) and significant thrombocytopenia (platelet count of 15,000/*μ*L) on admission, both of which remained low by Day 5. The white blood cell count was normal. Renal function and electrolyte levels were also within normal ranges throughout the observation period. C‐reactive protein was within the reference range, whereas D‐dimer was high and showed a rising trend during follow‐up (Table 1).

**TABLE 1:** Laboratory findings on admission and follow‐up.

Parameter	Day 1	Day 5	Reference range
Hemoglobin (g/dL)	7.8	7.7	12–15
Platelets (/*μ*L)	15,000	14,000	150,000–450,000
WBC (/*μ*L)	4200	4200	4000–11,000
Sodium (mmol/L)	143	144	135–145
Potassium (mmol/L)	4.3	4.0	3.5–5.0
Creatinine (mg/dL)	0.89	0.9	0.6–1.2
CRP (mg/L)	0.69	0.69	< 5
D‐dimer (ng/mL)	1330	1650	< 500

The high total bilirubin, lactate dehydrogenase, and reticulocyte count were consistent with hemolysis. The presence of schistocytes in peripheral blood smears supports the diagnosis of MAHA. The coagulation indicators were normal, and blood cultures were negative, helping to exclude disseminated intravascular coagulation and infectious etiologies, respectively.

Magnetic resonance imaging (MRI) of the brain showed no evidence of ischemic changes. Chest X‐ray and abdominal ultrasound were unremarkable.

The calculated PLASMIC score was 6, indicating a high probability of severe ADAMTS13 deficiency. Based on the high clinical suspicion of iTTP, laboratory findings, and PLASMIC score, treatment with corticosteroids (prednisolone) and therapeutic plasma exchange (plasmapheresis) was initiated without waiting for the ADAMTS13 results. Subsequent testing showed markedly reduced ADAMTS13 activity (< 5%), confirming the diagnosis of iTTP.

The patient also received one unit of packed red blood cells because of severe anemia, and a platelet transfusion because of severe thrombocytopenia and concern for sudden severe bleeding. Although platelet transfusion is generally not recommended in iTTP and is typically reserved for life‐threatening bleeding, the treating team decided to give platelets because of the multiple ecchymotic patches over the trunk and limbs in addition to the very low platelet count (15,000/*μ*L). The patient responded well to the treatment and demonstrated progressive improvement, with complete resolution of neurological symptoms and a steady rise in platelet count. She was discharged in stable condition after 2 weeks with hemoglobin 11.2 g/dL and platelet count of 194000/*μ*L. Arrangements were made for close outpatient follow‐up.

## 3. Discussion

iTTP is a medical emergency that requires prompt diagnosis and treatment due to its high mortality rate if treatment is delayed. In most adult cases, this deficiency is acquired and immune‐mediated, resulting from autoantibodies directed against ADAMTS13, which explains the predominance of acquired iTTP in clinical practice [2, 7]. The disease is rare, with an estimated annual incidence of approximately 2–6 cases per million population and shows a female predominance, occurring nearly twice in women compared to men [1, 2, 7].

In this case, the patient presented with transient neurological symptoms that were diagnosed as TIA. About 60% of iTTP patients may show neurological involvement as the most frequent organ manifestation, ranging from mild confusion and headache to focal deficits, seizures, or coma [4, 8]. The variable symptoms and signs in presentation cause diagnostic challenges, particularly when symptoms are transient and imaging studies are unremarkable, as observed in this case. Such transient neurological manifestations are generally attributed to cerebral microvascular ischemia resulting from platelet‐rich microthrombi.

In this case, the diagnosis of iTTP was supported by severe thrombocytopenia, MAHA (anemia, schistocytes, and elevated LDH), a normal coagulation profile, and a high PLASMIC score. The diagnosis was subsequently confirmed by markedly reduced ADAMTS13 activity. Importantly, treatment was initiated based on the clinical and laboratory findings and high PLASMIC score without waiting for the ADAMTS13 results, as treatment should not be delayed when iTTP is strongly suspected. It is worth noting that the classical presentation of the five features of iTTP that include fever, thrombocytopenia, MAHA, neurological symptoms, and renal dysfunction is uncommon as these are found together in less than 10% of the patients, indicating the need for updated diagnostic approaches [7]. The PLASMIC score can be used for predicting severe ADAMTS13 deficiency and guiding early treatment decisions [6]. A score of ≥ 6 indicates high probability and justifies immediate initiation of plasma exchange. In addition to neurological involvement, other organ systems may be affected due to microvascular ischemia. These include cardiac ischemia, mesenteric ischemia, and renal involvement [2, 8].

Therapeutic plasma exchange is the cornerstone of treatment, as it removes circulating autoantibodies and replaces ADAMTS13 [8, 9]. Corticosteroids are used to suppress autoantibody production. Current guideline‐based management of acute iTTP also supports the conditional addition of rituximab to suppress anti‐ADAMTS13 autoantibody production and reduce the risk of relapse, while caplacizumab, an anti‐vWF agent, is recommended to rapidly inhibit platelet‐vWF interaction and microvascular thrombosis [3, 8]. The use of these agents should be individualized according to the clinical setting, disease severity, patient‐related factors, and drug availability. Neither rituximab nor caplacizumab was administered in the present case; however, the patient responded favorably to plasma exchange and corticosteroid therapy. Intensive approaches such as twice‐daily plasma exchange or additional immunosuppressive agents may be required in refractory cases. Platelet transfusion is generally avoided in iTTP unless there is serious or life‐threatening bleeding because of concern for worsening microvascular thrombosis. In this case, platelet transfusion was administered because of severe thrombocytopenia and concern regarding the risk of severe bleeding, based on the treating team’s clinical judgment.

Early initiation of treatment significantly reduces mortality, which historically exceeded 90% but has now decreased to less than 20% with appropriate therapy. Despite these advances, iTTP continues to be in life‐threatening condition even with optimal management [2, 8]. It is worth noting that iTTP is characterized by a relapsing course, necessitating long‐term follow‐up for recurrence, associated autoimmune conditions, and possible long‐term complications. Monitoring ADAMTS13 activity during follow‐up may also help identify patients at increased risk of relapse and guide preemptive therapy.

## 4. Conclusion

This case shows that a TIA, most likely caused by early microvascular cerebral involvement, could be the first presentation of iTTP. Therefore, it should be considered in the differential diagnosis of patients presenting with transient neurological symptoms in association with hematological abnormalities, particularly anemia and thrombocytopenia. Early diagnosis is based on clinical features and laboratory findings, supported by the PLASMIC score and confirmed by severe ADAMTS13 deficiency. When clinical suspicion is high, treatment should be initiated promptly without waiting for ADAMTS13 results. Current management includes therapeutic plasma exchange, corticosteroids, and, where appropriate, rituximab and caplacizumab. Early recognition and appropriate treatment are critical for reducing morbidity and mortality and improving patient outcomes.

## Author Contributions

Hisham Eltayeb Ahmed Elhasan conceptualized the case report, provided the data, and drafted the manuscript. Fathelrahman Ahmed, Syed Majid Hamid, Tarig Merghani, and Azza Alawad reviewed and confirmed the patient data and edited the manuscript.

## Funding

No funding was received for this manuscript.

## Disclosure

All authors have read and approved the final version of the manuscript.

The corresponding author, Tarig Merghani, had full access to all of the data in this study and takes complete responsibility for the integrity of the data and the accuracy of the data analysis.

## Ethics Statement

Informed consent was obtained from the patient for publication of this case report.

## Conflicts of Interest

The authors declare no conflicts of interest.

## Supporting Information

Additional supporting information can be found online in the Supporting Information section.

## Supporting information


**Supporting Information** CARE‐checklist‐English‐2013 Filled.

## Data Availability

The data that support the findings of this study are available from the corresponding author upon reasonable request.
